# Validation of freeze-drier/plasma asher method for analysis of asbestos in the lung tissue

**DOI:** 10.3389/fchem.2025.1581910

**Published:** 2025-05-02

**Authors:** Anna Benedetta Somigliana

**Affiliations:** Electron Microscopy Laboratory, Environmental Protection Agency of Lombardy region, Milan, Italy

**Keywords:** asbestos, lung tissue, plasma asher, freeze drier, method validation

## Abstract

**Introduction:**

The analysis of asbestos fibers in lung tissue is complex due to both the biological matrix and the analyte. Lung tissue preparation techniques for asbestos burden analysis require the removal of organic matter to make the inorganic mineral components visible. The method’s validation is challenging due to the analyte’s inherent variability. This study outlines a procedure for validating an asbestos fiber analysis method in lung tissue.

**Methods:**

At the Electron Microscopy Laboratory of ARPA Lombardia, a method using a plasma asher for freeze-dried lung tissue digestion was developed. The analysis is carried out with a scanning electron microscope equipped with an energy-dispersive x-ray spectrometer. Problems associated with interferences, instrument calibration and resolution, analytical sensitivity, recovery were described in detail. The trueness and precision of the analytical method were evaluated using certified reference materials in accordance with ISO 33403:2024 standard.

**Results and conclusion:**

The developed method underwent a rigorous validation process to ensure metrological traceability of the results. Using validated analytical methods with consistent counting rules ensures comparability of data across laboratories while guaranteeing traceable results.

## 1 Introduction

The analysis of asbestos fibers in human lung tissue is crucial for understanding asbestos-related carcinogenic mechanisms and assessing past exposure when occupational history is unavailable.

This analysis presents significant challenges due to the complexity of the biological matrix, which requires extensive processing to render asbestos fibers measurable. Lung tissue preparation for asbestos burden analysis necessitates the removal of organic matter to isolate and visualize inorganic mineral components.

Numerous analytical methods have been documented in the literature for this purpose. The digestion of lung tissue can be achieved with chemical methods (for example, with hypochlorite or KOH), with low temperature oxygen plasma asher (LTA), but also with a combination of the two techniques. Several additional treatments, to complete tissue digestion, are described and the analysis can be carried out with a Scanning Electron Microscope (SEM) or with a Transmission Electron Microscope (TEM). Different counting rules are described in the literature, including the choice of asbestos fibers to be measured (Ashcroft and Heppleston, 1973; Gibbs et al., 1994; Gylseth et al., 1981; Karajalainen et al., 1993; Manke et al., 1987; Rogers, 1984; Roggli and Brody, 1984; Takahashi et al., 1994; Tuomi et al., 1989; William et al., 1982; Churg and Wood, 1983). The inherent variability of the lung tissue increases the overall uncertainty of this analysis (Churg and Wood, 1983; Morgan and Holmes, 1984; Gibbs and Pooley, 1996). This makes it more difficult to compare results from different laboratories (Gylseth et al., 1985). In 1998, a European Respiratory working group published specific guidelines on these subjects: “Guidelines for mineral fiber analyses in biological samples” (De Vuyst et al., 1998). Unfortunately, despite several contributions which clarified the basics needed to execute a reliable analysis, a standard method for this analysis had never been validated.

At the Electron Microscopy Laboratory, we developed a method for asbestos analysis in lung tissue using an oxygen plasma asher on lyophilized samples to “digest” organic tissue. The method has been developed as a result of numerous trials using both chemical and plasma asher digestion. In the end we opted for the plasma asher digestion of freed-dried lung tissues because it provides very efficient sample digestion, the ash is easy to process and there is the possibility of making several filters from a single incineration process. The methodological challenges related to counting rules were addressed in a previous study (Somigliana et al., 2023).

The development of any analytical method necessitates rigorous validation and among various international guidelines available for analytical method validation, the Eurachem Guide (Magnusson, 2014) was selected due to its comprehensive framework.

According to this guide:

“Method validation is basically the process of defining an analytical requirement and confirming that the method under consideration has capabilities consistent with what the application requires.

Inherent in this is the need to evaluate the method’s performance. The judgement of method suitability is important. The validation work is preceded by a development phase which may involve different staff, and which can take a number of forms”.

Validating this type of analysis is particularly complex due to the inherent variability of the analyte.

In standard chemical or physical analyses, the measurand is typically a single molecule or a parameter with well-defined chemical and physical characteristics.

In contrast, asbestos fiber analysis involves six distinct mineral types, each with different physical properties. Fiber dimensions and morphologies vary widely, from single fibrils (∼30 nm in diameter) to fiber bundles exceeding tens of micrometers (Baron, 2001). Additionally, asbestos fiber lung distribution differs between individuals and evolves over time due to lung clearance mechanisms and structural breakdown of fiber bundles.

The conventional concept of “recovery” in chemical analysis is particularly difficult to apply in this context.

Typically, recovery is assessed using fortified matrices—spiked samples with known analyte concentrations, however, the heterogeneous nature of asbestos fibers complicates this approach, necessitating alternative validation strategies.

Creating a standardized solution to fortify lung tissue samples with asbestos in a laboratory setting presents significant challenges. Such a solution would require a precisely controlled asbestos fiber distribution, which is inherently difficult to achieve. Even if such a solution were available, the variability of the analyte would make an adequate, statistically significant recovery assessment impractical due to the extensive number of measurements required.

Beyond recovery determination, analytical method validation must also verify the following parameters:

• Interference and instrumental resolution (the minimum size of detectable and countable asbestos fiber).
• Analytical sensitivity (AS) and working range.
• Precision (repeatability and reproducibility).
• Trueness (linked to the concept of “recovery” - bias).
• Measurement uncertainty.

This study details the development of an analytical method for asbestos detection in lung tissue and its validation process.

## 2 Methods

### 2.1 Analytical method

Lung tissue, typically stored in formalin, undergoes a preparatory process before analysis. About 1–3 cm^3^ of lung tissue is first immersed in filtered double-distilled water to remove formalin. If the lung tissue is available, several fragments taken from different parts of the lung are lyophilized. The lung is then frozen at 255 K and immersed in liquid nitrogen to reach a temperature of about 77 K. The lung tissue is then freeze-dried for 72 h (Labconco Freezone 6). To ensure representative sampling, 100 mg of dry tissue is collected from various regions of the lyophilized lung and subjected to incineration using an oxygen plasma asher for 24 h at 60–80 w (Diener Electronic GmbH, Pico). The application of oxygen plasma asher to freeze-dried samples was first proposed by Manke et al. (1987). The resulting ash is suspended in 100 mL of double-distilled water, manually shaken and filtered through a polycarbonate 25 mm diameter membrane with 0.2-μm porosity, allowing for the creation of multiple filters with increasing ash loads. The active filtration area of our filtration system is 2.22 cm^2^. If the plasma asher works well, no ultrasonic treatment is necessary. To ensure complete digestion of organic residues, 1–2 mL of 8% oxalic acid may be added before filtration. This process typically yields filters with an ash load equivalent to 20 mg of dry lung if 20 mL of solution is filtered. In this case the ash load of the filter is about 9 mg of dry lung per cm^2^. If the lung tissue is not excessively charged with particulate matter, filters with a concentration up to 15 mg of dry lung per cm^2^ may be analyzed.

The filters are then metallized with platinum, gold or gold/palladium (Quorum Technologies Sputter Coater Q150 R Plus) and analyzed using a field emission scanning electron microscope Zeiss Sigma 300 equipped with an energy-dispersive x-ray spectrometer Oxford Ultim Max (SEM-EDS).

The asbestos fiber counting methodology follows the same principles as airborne asbestos fiber analysis on membrane filters. Accordingly, its performance characteristics align with reference methods such as RTM2 (1984), ISO 14966 (2019), and ISO 13794 (2019) (Asbestos International Association, 1984; ISO and ISO 14966, 2019; ISO and ISO 13794, 2019). The filter must be homogeneous, free of localized particles accumulations or deposition-free areas, Otherwise, unfortunately it must be reprepared.

### 2.2 Counting rules

All asbestos fibers longer than 1 μm are counted at ×12,000 magnification, with results expressed as the number of fibers >1 μm per gram of dry tissue (ff/g dry). By knowing the lung tissue load deposited on the filter, it is possible to calculate the area of the filter that needs to be analyzed in order to obtain the analytical sensitivity required for the analysis. The analysis ends when this area is analyzed or when 50 fibers are counted, even in a lower area.

### 2.3 Instruments calibration

All instruments used for measurement, including scales for determining sample weight, were calibrated by accredited laboratories recognized under international mutual recognition agreements, such as those based on ISO/IEC 17025 (ISO and ISO/IEC 17025:2017, 2017). Additionally, select measurement parameters were internally calibrated using certified reference materials (CRM), such as the microscope’s field area at ×12,000 magnification and the ruler for microscopic dimension measurement.

### 2.4 Blanks

All reagents shall be tested to exclude possible asbestos contamination. In addition, the whole procedure must be carried out without lung tissue: an empty container must undergo to the same preparation of the lung. This check should be repeated periodically to assess the effects of the laboratory environment on the particulate load on the filter.

### 2.5 Recovery

Recovery was assessed indirectly by verifying that the lung tissue preparation method does not alter the composition or morphology of the contained asbestos fibers.

To evaluate the effect of the preparation technique, lung tissue spiked with asbestos was used. A sample of asbestos-free lung tissue was injected with approximately 1 mL of an aqueous solution containing the three primary commercial asbestos varieties: chrysotile, amosite, and crocidolite. This solution was prepared by finely grinding small quantities of commercial asbestos (NIST 1866b) with salt and ethyl alcohol in a mortar. The resulting powder was suspended in 50 mL of distilled water and filtered through high-porosity paper to remove the larger fibres (∼20 μm). This is done in order to obtain a fiber size distribution which approximates airborne asbestos fibers distribution. Excessive grinding was avoided to preserve the crystalline structure of the fibers.

A 1-mL aliquot of this solution was filtered onto a 0.2-μm porosity polycarbonate membrane and analyzed using SEM-EDS to obtain images and X-ray spectra of unprocessed asbestos fibers. These data were then compared with images and spectra from the fibers in the spiked lung sample.

The fortified sample was also utilized to assess potential interferences and determine the instrument’s resolution, specifically the minimum asbestos fiber diameter detectable by the SEM-EDS employed in this study.

### 2.6 Trueness and precision

The trueness and precision of the analytical method were evaluated using certified reference materials BCR-665 and BCR-666, which consist of lyophilized and homogenized lung tissue with certified concentrations of amphibole asbestos fibers (amosite + crocidolite) and anthophyllite (Tossavainen et al., 2001). Results are expressed as millions of fibers per gram of dry tissue.

Unfortunately, there is no lung tissue reference material available that is certified for chrysotile asbestos content. For this reason, it is not possible to carry out a similar precision and trueness assessment for chrysotile asbestos. However, the recovery assessments above described carried out on chrysotile asbestos, reasonably allow to extend the use of the method to this type of asbestos.

Different batches of these reference materials were analyzed eight times, and the results were assessed in accordance with ISO 33403 (2024) standard (ISO and ISO 33403, 2024).

### 2.7 Analytical sensitivity and working range

Analytical sensitivity was defined as the concentration corresponding to the detection of a single asbestos fiber in the analysis. The working range was determined through calculation.

### 2.8 Measurement uncertainty

Measurement uncertainty can be assessed using various approaches. If the precision and trueness align with those provided for BCR-665 and BCR-666, the uncertainty values from the CRM certificate can be applied.

## 3 Results

The SEM-EDS, operating at ×12,000 magnification, enables the visualization and identification of fibrils as small as ∼30 nm in diameter.

A comparative analysis of asbestos fiber images and X-ray spectra from the aqueous solution and the fortified lung tissue demonstrated that the lung tissue preparation method does not significantly alter the morphology or X-ray spectra of asbestos fibers.

**FIGURE 1:**
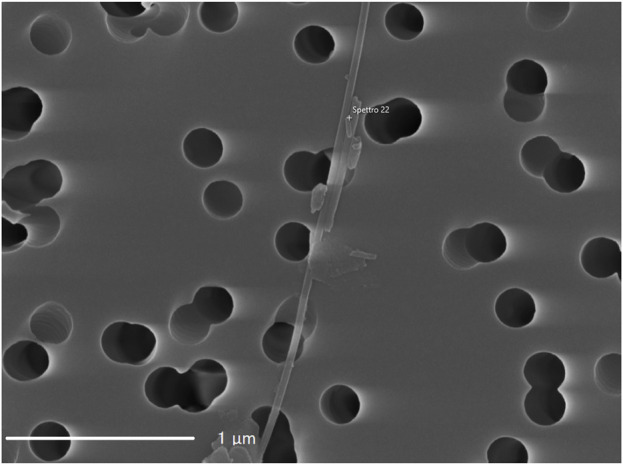
Image of NIST 1866b chrysotile fibril from the water solution.

**FIGURE 2:**
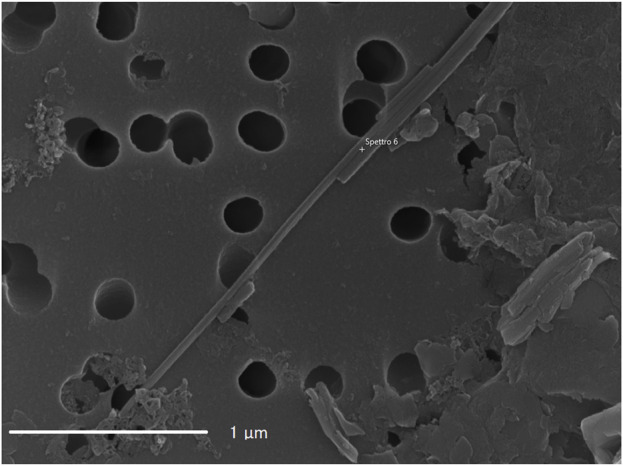
Image of NIST 1866b chrysotile fibril from the spiked sample.

**FIGURE 3:**
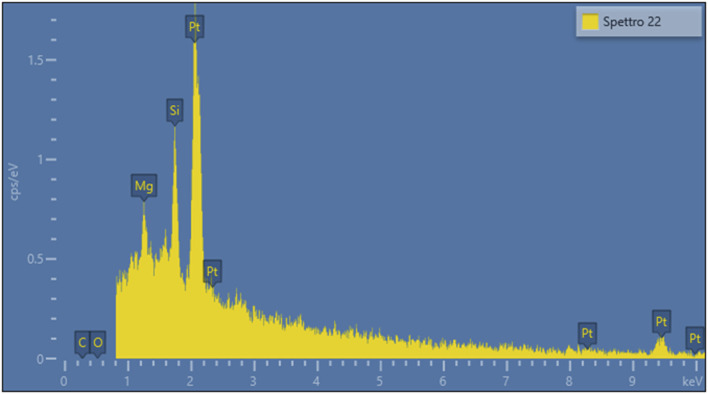
X-ray spectrum of the fibril in Figure 1.

**FIGURE 4:**
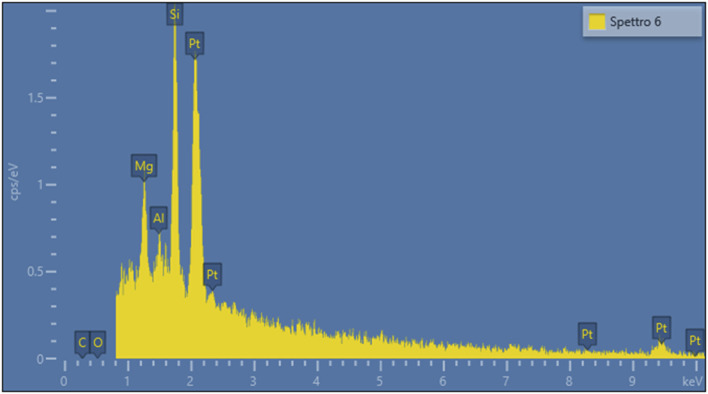
X-ray spectrum of the fibril in Figure 2.

Figures 1, 2 present representative images of two chrysotile fibrils (∼30 nm in diameter) from both the spiked lung tissue and the untreated solution, along with their respective X-ray spectra (Figures 3, 4).

In microscopy analysis, the entire filter is typically not examined. In a homogeneous sample, the results from a portion of the filter can be extrapolated to the entire membrane using a proportional calculation, provided that an equiprobable sampling method is applied.

Consequently, the concentration of asbestos fibers in lung tissue is determined using the following formula:

$$C\;\left(ff/gdry\right)=\frac{n_{f}\times A}{N_{fields}\times a\times p_{TS}}$$

where,

*n*_*f*_ is the number of asbestos fibers counted in the analysis.

*A* is the effective filtration area of the filter (mm^2^).

*N*_*fields*_ is the number of microscopic fields analyzed.

*A* is the area of the microscopic field at ×12,000 magnification (mm^2^).

*p*_*TS*_ is the weight of the lung tissue on the filter.

The uncertainty component of the counting method is derived from the Poisson distribution. Therefore, for *n* fibers counted during analysis, the standard deviation of the count is expressed as √n.

Both analytical sensitivity (AS) and working range are determined through calculation.

The AS represents the concentration corresponding to the detection of a single asbestos fiber in the analysis and should be at least 0.1 mil ff/g dry.

The working range spans from 0 to approximately 300 mil ff/g dry, although it can be extended by analyzing a filter with a lower lung tissue load.

For assessing trueness and precision, reference materials BCR-665 and BCR-666 were utilized. Table 1 summarizes the measurement results for these certified reference materials (CRMs), along with the formulas and calculations used to evaluate precision and trueness.

**TABLE 1:** Precision and trueness verification according to ISO 33403:2024 standard.

	BCR-665 Amosite + crocidolite (mil ff/g dry)	BCR-666 Anthophyllite (mil ff/g dry)
x_meas;BCR_	49.6	5.8
x_BCR_	49.0	5.1
s_w;BCR_	7.5	0.92
σ_w0;BCR_(Poisson)	7.0	0.72
u_meas;BCR_	7.4	0.87
U_BCR_	16	1.5
$u_{\text{BCR}}=\frac{U_{\text{CRM}}}{t_{\left(p‐1\right);0.95}}*$	$\frac{16}{2.45}=6.53$	$\frac{1.5}{2.45}=0.61$
${Χ}_{c;BCR}^{2}={\left(\frac{s_{w;BCR}}{\sigma_{w0;BCR}}\right)}^{2}$	1.15	1.56
${Χ}_{table}^{2}=\frac{\chi_{\left(n-1\right);95\%}^{2}}{n-1}$	2.01	2.01
Precision	${Χ}_{c;BCR}^{2}=1.15<{Χ}_{table}^{2}=2.01$	${Χ}_{c;BCR}^{2}=1.56<{Χ}_{table}^{2}=2.01$
$\left|x_{meas;BCR}-x_{BCR}\right|$	0.6	0.7
$k\sqrt{u_{meas;BCR}^{2}+u_{BCR}^{2}}$	19.7	2.26
Trueness	$\left|x_{meas;BCR}-x_{BCR}\right|=0.6<k\sqrt{u_{meas;BCR}^{2}+u_{BCR}^{2}}=19.7$	$\left|x_{meas;BCR}-x_{BCR}\right|=0.7<k\sqrt{u_{meas;BCR}^{2}+u_{BCR}^{2}}=2.26$

x_meas;BCR_: arithmetic mean of the 8 measurements of the CRMs.

x_BCR_: certified reference value.

s_w;BCR_: standard deviation of the measurements.

σ_w0;BCR_ (Poisson): is the specified value of the intralaboratory standard deviation, the Poisson standard deviation was used as the reference value.

u_meas;BCR_: standard uncertainty of the measurement result.

U_BCR_: uncertainty of the certified value: the uncertainty is the 95% confidence interval of the mean of laboratory mean values.

*u_BCR_: calculated from U_BCR_, as indicated in ERM, Application note 1 (ERM Application note 1, 2010).

Χ^2^_table_:: denotes the 0,95th quantile of the χ2 distribution with (n-1) degrees of freedom, divided by the degrees of freedom (n-1).

No evidence suggests that the method’s precision deviates from expectations based on the Poisson uncertainty component. For both reference materials, the measured mean values were not significantly different from the certified values.

Upon analyzing the various components of the overall uncertainty, it becomes evident that the Poisson component, linked to the counting method, is the dominant source.

The extended measurement uncertainty provided by the CRMs is approximately 30%, expressed as a percentage coefficient of variation.

When 50 fibers are counted, the standard Poisson uncertainty, expressed as a percentage coefficient of variation, is given by:

$$\sqrt{n/n}*100=\sqrt{50/50}*100≅14\%$$

The extended Poisson uncertainty is 28%, which closely aligns with the overall uncertainty provided for the CRMs.

## 4 Discussion and conclusion

Analyzing asbestos in lung tissue is inherently challenging due to the complexity of both the analyte and the biological matrices involved.

Since no standardized method existed before this work, the developed method underwent a rigorous validation process to ensure metrological traceability.

The decision to count only asbestos fibers >1 μm was based on the certification of the two available reference materials for amphibole fiber content >1 μm.

It is important to note that using validated analytical methods, with consistent counting rules, ensures comparability of data across laboratories while guaranteeing traceable results.

In conclusion, this study outlines a procedure for validating an asbestos fiber analysis method in lung tissue, leading to accreditation of the Electron Microscopy Laboratory under ISO 17025 for the described method.

## Data Availability

The raw data supporting the conclusions of this article will be made available by the authors, without undue reservation.
